# Supramolecular exfoliation of layer silicate clay by novel cationic pillar[5]arene intercalants

**DOI:** 10.1038/s41598-021-90122-9

**Published:** 2021-05-20

**Authors:** Takahiro Kakuta, Yudai Baba, Tada-aki Yamagishi, Tomoki Ogoshi

**Affiliations:** 1 Graduate School of Natural Science and Technology, Kanazawa University, Kakuma-machi, Kanazawa, 920-1192 Japan; 2 WPI Nano Life Science Institute (WPI-NanoLSI), Kanazawa University, Kakuma-machi, Kanazawa, 920-1192 Japan; 3 Graduate School of Engineering, Kyoto University, Katsura, Nishikyo-ku, Kyoto, 615-8510 Japan

**Keywords:** Nanocomposites, Supramolecular chemistry

## Abstract

Clays are multi-layered inorganic materials that can be used to prepare nanocomposite fillers. Because the multi-layered structure is thermodynamically stable, it is difficult to change a multi-layered material into single layers to improve its dispersity. Previously, clays were modified with dodecylammonium cations to promote complexation with nylon 6, nylon 66, polypropylene, polyethylene, polystyrene, and polycaprolactone to increase the mechanical strength (and/or thermal stability) of the composite material; however, complete exfoliation could not be achieved in these composites. In this study, pillar[5]arenes are synthesized and functionalized with ten cationic substituents as novel intercalants for modifying bentonite clay, which is a multi-layered metal-cation-containing silicate. The pillar[5]arenes exfoliate the clay by forming polyrotaxanes with poly(ethylene glycol) through host–guest interactions.

## Introduction

In natural systems, layered materials, such as phospholipid bilayers, maintain stacking through intermolecular interactions^1–3^. These materials are not only used as structural components for imparting mechanical strength in composites, but also for their specific functions, such as providing ion transport via transport proteins, which has been observed in various multilayer systems. Generally, multilayered materials are constructed using the same components for each layer. For example, multi-walled carbon nanotubes and graphite comprise stacked graphene films, while natural clays possess layered silicate sheets^4–9^. It is difficult to change the structure of a multilayered material into a single layer because the multilayered structure is thermodynamically stable^10^. Therefore, the exfoliation of each layer of a multilayered material requires external stimuli, such as high temperatures and/or destructive forces^11^. Single-layered materials exhibit specific properties, including liquid crystalline states^12–14^ and high conductivities^15^, that result from their large surface areas and high aspect ratios. To the best of our knowledge, methods for preparing single-layer clays with potential multifunctionality have not received sufficient consideration.

Clays are multilayered inorganic materials that can be used as nanocomposite fillers because they are highly thermally and mechanically stable and impart this stability to composites of which they are constituents^16,17^. The inclusion of single-layered, highly dispersed clay in a polymer matrix can exploit the properties of the clay as a structural component that efficiently endows the polymer with the silicate-, aluminum-, and magnesium-based properties of the clay. In addition, single-layered clays have large surface areas and are good sorbents^18^. To prepare a single-layered clay, an alkyl-group-containing cation is often induced as an intercalant into the interlayer spaces of the clay to promote exfoliation. For example, montmorillonite has previously been modified with the dodecylammonium cation for its subsequent complexation with various polymers, such as nylon 6, nylon 66, polypropylene, polyethylene, and polystyrene, to increase their strengths^11,19^.

However, the organoclays could not be completely exfoliated in these composites. Thus, it is difficult to change the structure of a clay to achieve single layers, even when its dispersity is enhanced with a cationic intercalant. Okamoto and co-workers reported that bentonite, which is a multilayered silicate containing metal cations, could be perfectly exfoliated in nylon 6^20,21^. They reported that high temperature and pressure were required as high-energy sources to separate the layers. Additionally, Akita and co-workers^22^ and Brito et al.^23^ found that centrifugation and annealing processes were necessary to achieve a single-layered clay. Clearly, perfect exfoliation methods are poorly understood, although several mechanisms have been proposed. With this in mind, the development of a new method for the exfoliation of clay remains a challenge in the field of composite materials.

**Figure 1:**
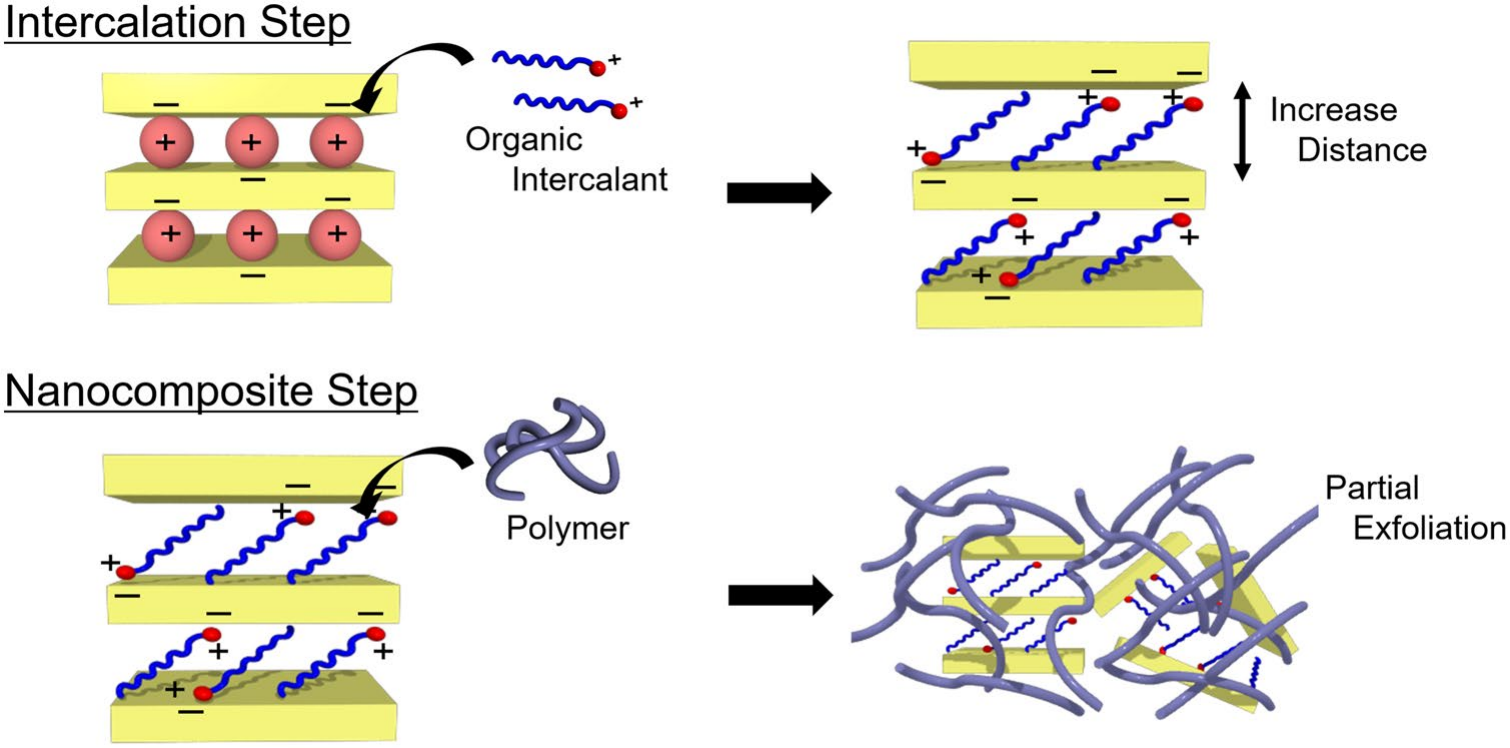
Schematic illustration of a polymer–clay nanocomposite formed using the intercalation method. In the intercalation step, organic intercalants penetrate the interlayer space. The internal cations are exchanged for the organic intercalants because they have higher affinities for the silicate layers than for the cations. The interlayer distance is increased after intercalation. Subsequently, polymer molecules penetrate the interlayer space of the modified clay, which has partially separated in the polymer matrix.

**Figure 2:**
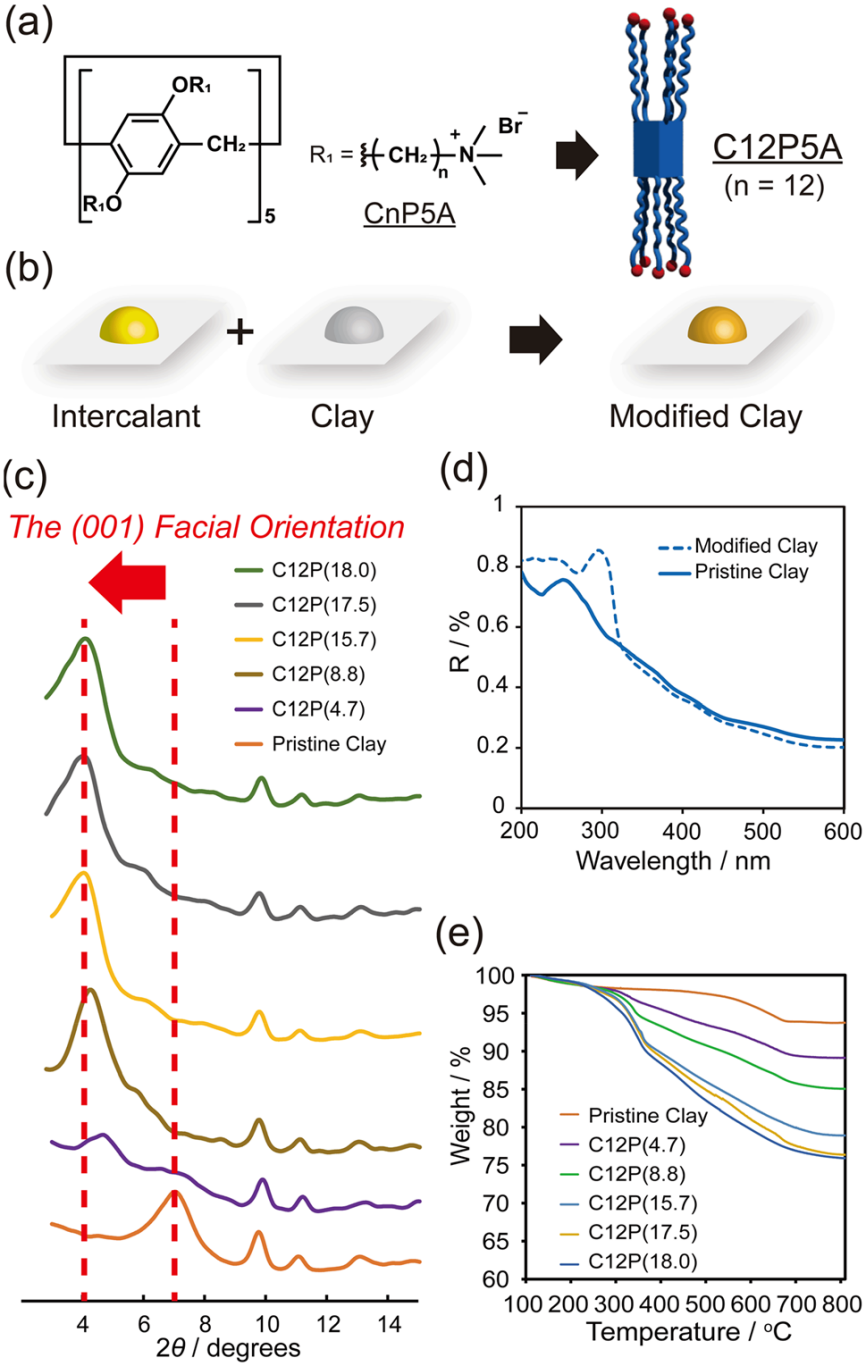
Intercalating C12P5A into bentonite clay. (**a**) Chemical structure of cationic pillar[5]arene. (**b**) Preparation of a modified clay. Differently colored clays were obtained after modification. (**c**) Powder X-ray diffraction (PXRD) patterns of the modified clays (C12P(X), where X denotes the percentage of C12P5A loaded in the clay). The peak associated with the interlayer distance increased with an increase in the amount of C12P5A. (**d**) UV–Vis absorption spectra of the pristine and modified clays. The peak for the benzene ring at 295 nm was only observed for the modified clay. (**e**) Thermogravimetric analysis (TGA) traces for calculating the amount of C12P5A.

A typical method for the preparation of a polymer–clay nanocomposite is shown in Fig. 1. Interlayer cations, which stabilize layered silicates by forming ion pairs with anionic compounds, are exchanged for organic intercalants that are better able to acclimate to the polymer matrix. In this method, perfect exfoliation is difficult because only weak capillary forces act to insert the polymer molecules between the clay layers. Therefore, adding another selective intermolecular interaction, such as the host–guest interaction of a pillar[n]arene, may be a valid method for increasing the polymer-penetration energy. A pillar[n]arene is a cyclic molecule in which n benzene rings are connected through methylene linkages at their *para* positions^24,25^. A previous study reported that crystalline pillar[5]arenes form polypseudorotaxanes with polymers in the bulk state^26^. Herein, we synthesized pillar[5]arenes functionalized with ten cationic substituents (CnP5A, where n denotes the number of carbons in the substituent group; see Fig. 2a) as novel intercalants that operate through host–guest interactions for the exfoliation of bentonite clay. The CnP5A-modified clays show greater interlayer distances than those of the pristine clay and improved affinities toward polymer matrices. They also achieved perfect exfoliation through host–guest interactions between pillar[5]arene and poly(ethylene glycol) (PEG). The small amount of energy associated with the formation of inclusion complexes in this method is smaller than those of previous methods and is used to facilitate the exfoliation of the clay. Our novel exfoliation system yields polymer-based composite materials containing highly dispersed single-layered clays.

## Results

### Preparing surface-modified clays

Commercial bentonite clay has a layered silicate structure that contains a sodium cation at the center of each silicate unit. We intercalated bentonite by stirring it with C12P5A at 80 °C in water for 18 h to exchange its sodium cations. When C12P5A and bentonite were mixed in a 1:1 (w/w) ratio, the modified clay was pigmented, whereas the pristine clay was not (Fig. 2b). The modified clay was investigated by powder X-ray diffraction (PXRD) (Fig. 2c). The PXRD pattern revealed a peak at 2*θ* = 7.0° (*d*_001_ = 12.6 Å) for the pristine clay. This peak is attributed to the (001) facial orientation and the interlayer distance of the silicate^27^. The peak observed at 2*θ* = 4.0° (*d*_001_ = 22.1 Å) in the PXRD pattern of the modified clay (Fig. 2c, green) suggests that the sodium cations were exchanged for C12P5A without exfoliating the clay. In addition, the modified clay without intercalants exhibited a PXRD pattern similar to that of the pristine clay, which reveals that introducing the intercalant between the clay interlayers of the C12P5A-modified clay changes its PXRD pattern. The presence of C12P5A in the clay was confirmed by solid-state UV–vis absorption spectroscopy (Fig. 2d). The modified clay exhibited a band at 302 nm, which is not observed in the spectrum of the pristine clay. Although the clay itself does not absorb in the UV region, pillar[5]arene absorbs at 295 nm because of its benzene rings. Because the modified clay was washed with sufficient water and methanol to remove residual C12P5A, the C12P5A present in the modified clay must be present in the interlayer spaces.

The percentage of C12P5A in the feed was changed from 5 wt% to 50 wt% in order to investigate the intercalation of C12P5A in the interlayer of the clay. The amount of C12P5A in the modified clay (*IC*) was determined by thermogravimetric analysis (TGA) according to

1 $$IC={R}_{c}-{R}_{dc},$$

where *R*_*c*_ and *R*_*dc*_ are the residual amounts of pristine clay and modified clay, respectively (Fig. 2e). Based on the TGA measurements, C12P5A exhibits a thermal decomposition point at 180 °C with 0 wt% residual amounts at 560 °C. *IC*, *R*_*c*_, and *R*_*dc*_ are summarized in Table S1, which reveals that the modified clay contains 18.0 wt% C12P5A when mixed in a 1:1 ratio (50 wt%). Meanwhile, modified clays prepared under 5, 10, 20, and 30 wt% conditions contain 4.7, 8.8, 17.5, and 15.7 wt% C12P5A, respectively. Since the error was 2 wt%, the mixing ratio was saturated at 20 wt%. Each modified clay is referred to as “C12P(X)”, where X is the percentage of C12P5A loaded in the clay. A comparison of the C12P(4.7) and C12P(18.0) PXRD data (using a 1.54-Å X-ray source) reveals that the *d*_001_ value increases with increasing X (Fig. 2c). We suggest that this is due to the exchange between the sodium cations and C12P5A, which increases the interlayer distance because C12P5A is larger than the sodium cation.

**Figure 3:**
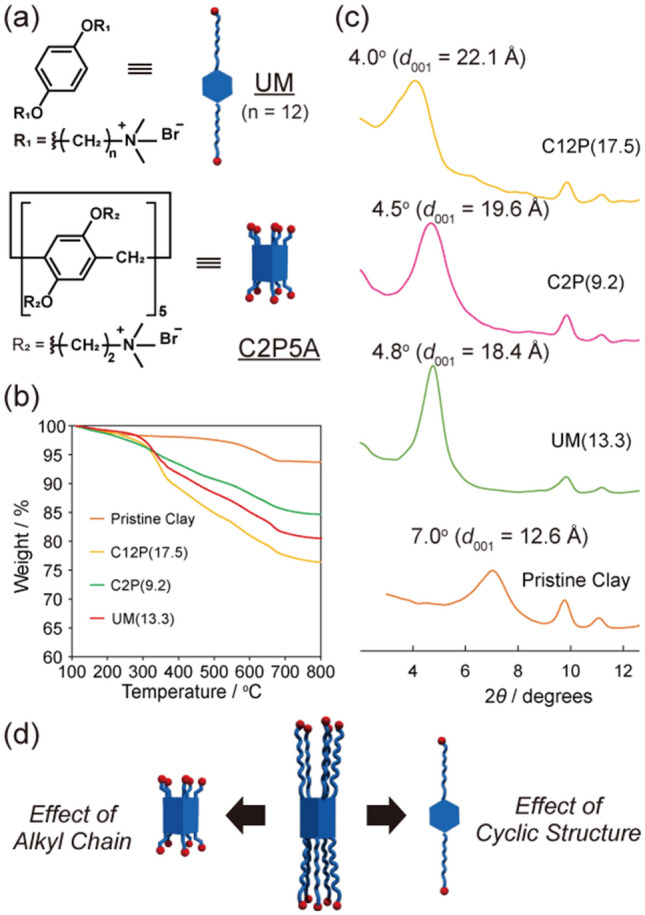
Effect of chain length and cyclic structure on clay modification. (**a**) Chemical structures of the model unit (UM) and C2P5A. (**b**) TGA traces of pristine clay, C12P(X), C2P(X), and UM(X) used to calculate their included amounts of C12P5A. (**c**) PXRD patterns for comparing intercalants. The peaks reveal different interlayer distances due to the intercalants. (**d**) Schematic of effective intercalation using C12P5A.

Alkylammonium cations have previously been shown to be useful intercalants for clay–polymer nanocomposites because their long alkyl chains easily interact with the layered structural components and are retained there. The C12P5A intercalant structure contains ten long alkyl chains and ten ammonium cations. Consequently, it interacts with anionic clay surfaces more strongly than previously used intercalants, and therefore it intercalates more efficiently. To investigate the effect of cationic pillar[5]arene encapsulation in the clay, we prepared two modified clays, one with the C12P5A molecular unit (UM), and the other with C2P5A, which contains shorter alkyl chains than those of C12P5A (Fig. 3a). Subsequently, TGA (Fig. 3b) revealed that the C2P(X) and UM(X) modified clays contained 9.2 wt% C2P5A and 13.3 wt% UM, respectively, when mixed at a 20 wt% feed ratio (Table S1). The PXRD patterns of these modified clays are shown in Fig. 3c. These patterns enabled us to compare the interlayer distances of the clays. The (001) peak of C2P(9.3) showed a slight shift to a smaller angle (2*θ* = 4.5°; *d*_001_ = 19.5 Å) compared to that of the pristine clay. Similarly, the UM-modified clay (UM(13.3)) exhibited a 2*θ* value of 4.8° (*d*_001_ = 18.4 Å). When compared with C12P(17.5), it was clear that the long alkyl chains and cyclic structure of C12P5A were necessary factors for its effective modification. The penetration mobility of the long alkyl-chain-containing cationic groups of C12P5A in the clay interlayers is clearly higher than that of the short chains in C2P5A (Fig. 3d). In addition, compared to UM, the highly symmetric structure of the pillar[5]arene works to effectively modify clay because it can easily aggregate^28–31^.

**Figure 4:**
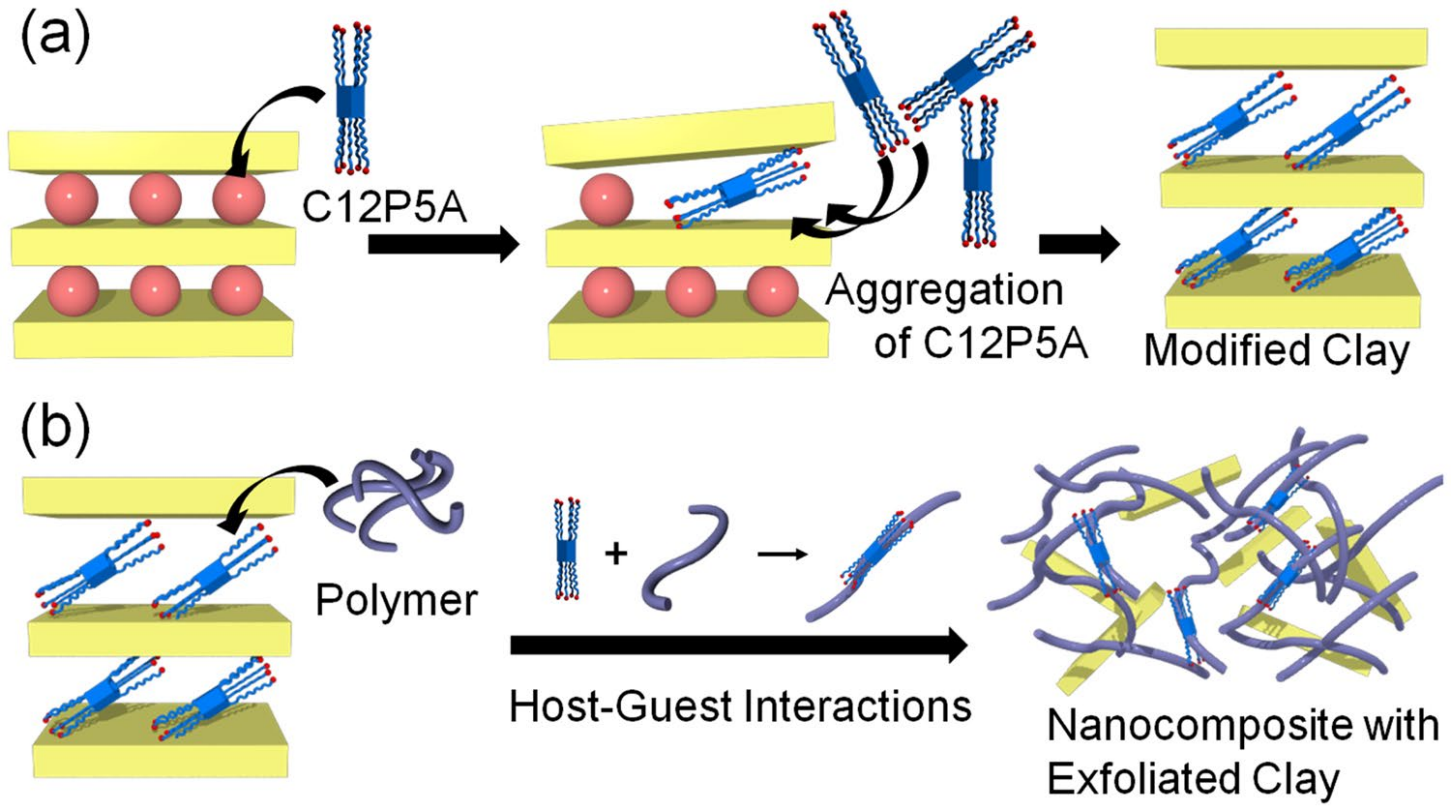
Proposed mechanism. (**a**) Clay intercalation using C12P5A and (**b**) clay exfoliation through host–guest interactions between C12P5A and poly(ethylene glycol) (PEG) within the interlayer.

Based on these results, we propose a mechanism for the intercalation of C12P5A into bentonite, as shown in Fig. 4a. Alkylammonium cations on the pillar[5]arene rim approach the clay interlayer and exchange with the sodium ions. Because C12P5A has ten alkyl cations, it is easily exchanged with the interior cations through strong interactions between the clay layers and its cationic moieties. The introduced intercalant then aggregates on the silicate layer, aided by its symmetrical structure, with the stacked C12P5A expanding the interlayer distance.

### Exfoliating modified clay by mixing with PEG

**Figure 5:**
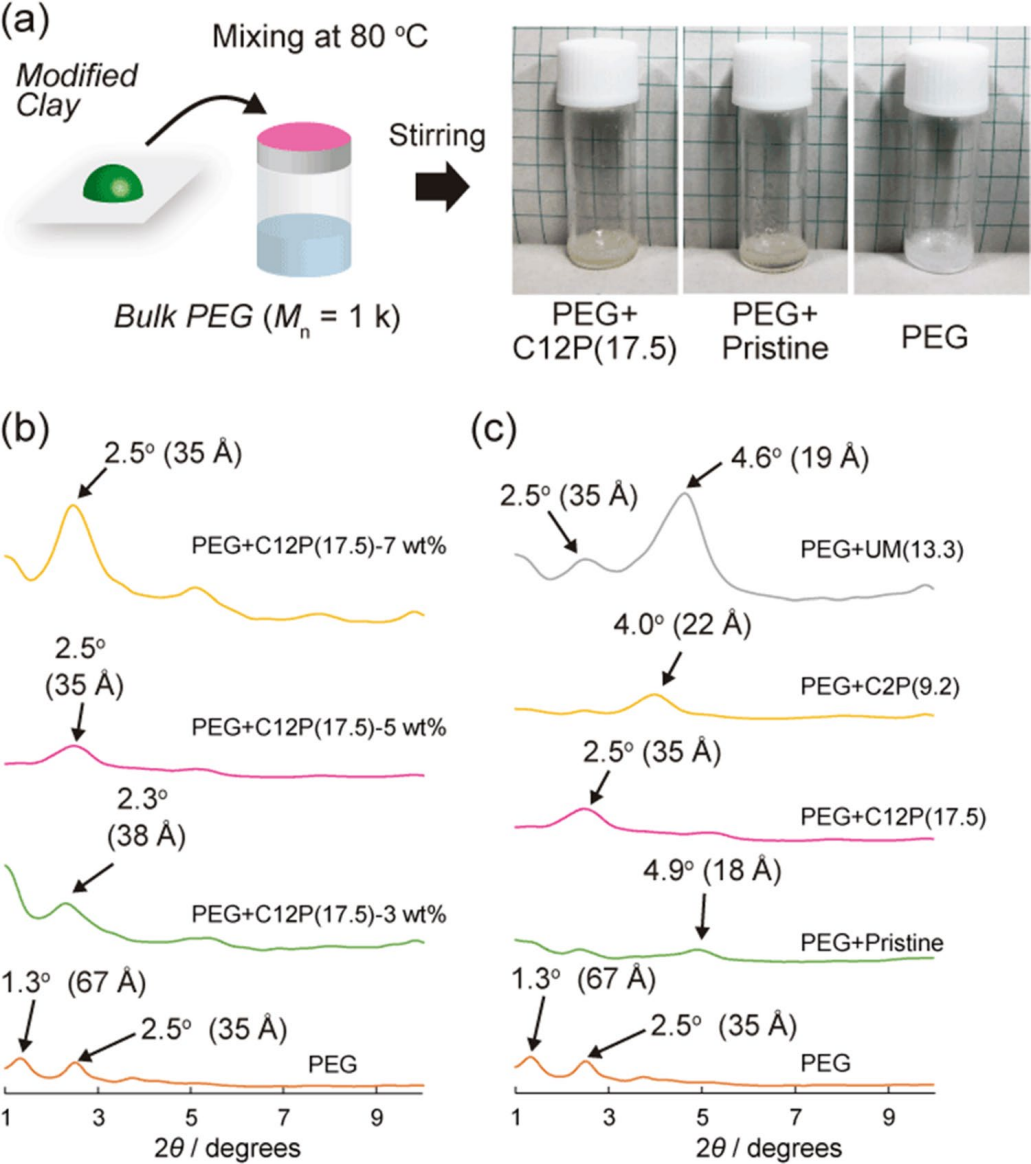
Clay in the PEG matrix. (**a**) Preparing a nanocomposite with modified clay. PXRD patterns of PEG with (**b**) various amounts of added clay and (c) various intercalants.

Modified clays can be exfoliated by compositing with polymer matrices under specific conditions, such as high temperatures and/or high pressures. However, freely combining polymers, intercalants, and clays is difficult. Therefore, examples of perfect exfoliation are limited. In particular, PEG, which is a low-toxicity, highly hydrophilic polymer, can be usefully composited with single-layered clays^32,33^. Supramolecular interactions, such as host–guest interactions involving pillar[5]arenes, yield inclusion complexes with PEG in the bulk state^26^. Host–guest interactions are expected to strongly assist the dispersion of a single-layered clay in a PEG matrix. Because modified clays are stable at high temperature, mixtures of modified clays and PEG (95/5, w/w) were stirred in the bulk state for 24 h at 80 °C, which exceeds the melting point of PEG (*M*_n_ = 1 k) (Fig. 5a). The PEG + C12P(17.5) mixture was a uniformly yellow solid at room temperature. In contrast, the color of the pristine clay in PEG (PEG + pristine) was slightly yellow and the clay was noted to be precipitated from the PEG. Hence, we concluded that modified clays are highly dispersed in the PEG matrix.

To investigate the interlayer structure of the modified clay in the PEG matrix following mixing, the composite material was subjected to PXRD under same experimental conditions (Fig. 5b). The spectrum of PEG + C12P(17.5) only exhibited a peak at 2*θ* = 2.5° (*d*_001_ = 35 Å) that corresponds to the (001) facial orientation, and this peak angle is smaller than that observed for C12P(17.5) (2*θ* = 4.0°). In addition, when pristine clay was complexed with PEG in the bulk state, the (001) peak was observed at approximately the same angle (2*θ* = 4.9°; *d*_001_ = 18 Å). PEG also exhibits diffraction peaks because it is a semi-crystalline polymer. The intensity of the 2*θ* peak at 2.5° in the PXRD spectrum of PEG + C12P(17.5) was observed to be as small as that of the peak corresponding to PEG. Furthermore, the PXRD spectra of other modified clay samples prepared by mixing PEG and C12P(17.5) at 3 and 7 wt% also showed the same peak (2*θ* = 2.5°; *d*_001_ = 35 Å). Therefore, we concluded that PXRD from the clay interlayer in PEG + C12P(17.5) effectively disappeared upon mixing with PEG.

To investigate the clay structure inside the PEG matrix, we evaluated this composite material by small-angle X-ray scattering (SAXS). Figure S7 and S8 show Guinier plots of PEG + C12P(17.5) and pristine PEG. The SAXS profile of PEG + C12P(17.5) was fitted using the Guinier approach^34^ according to the following equation:

2 $$I\left(q\right)={I}_{o}\mathrm{exp}\left(\frac{-{R}^{2}}{3}{q}^{2}\right),$$

where *q* is the scattering vector and *R* is the Guinier radius, which is a measure of the size of the scattering object (particle size) (Table 1). The scattering intensity of PEG + C12P(17.5) was determined to be the same as that of pristine PEG. By paying attention to the value of *R*, the height (*H*) of the clay, as a cylindrical model, in the composite material can be calculated using the following equation:^35^

**Table 1:** PXRD data and small-angle X-ray scattering (SAXS) Guinier-plot data for clays in PEG.

Entry	Composite Material *^a^	Intercalant	Induced Ratio [/wt%]	Interlayer Distance*^b^ [(*d*_001_) / nm]	Δ *d*_001_ *^c^ [/nm]	Guinier Radius (*R*) *^d^	H*^d^	Aspect Ratio
1	PEG + C12P(17.5)	C12P5A	17.5	22.1	9.5	5.4	1.2	16.1
2	PEG + C2P(9.2)	C2P5A	9.2	19.5	6.9	5.7	5.5	3.4
3	PEG + UM(13.3)	UM	13.3	18.4	5.8	6.2	8.5	2.2
4	Ideal Monolayer Clay	–	–	12.6	–	–	1.0	18.7

*^a^ Clay additive condition is similar at 5 wt%; *^b^ Interlayer distance of modified clays obtained by PXRD; *^c^ Δ*d*_001_ = (*d*_001_ of modified clay) − (*d*_001_ of pristine clay); *^d^ Guinier radii and *H* values were calculated according to a previous report^35^.

3 $$R=\sqrt{\frac{{D}^{2}}{8}+\frac{{H}^{2}}{12},}$$

where *D* is the diameter of the model cylinder. The *H* value of PEG + C12P(17.5) was determined to be 1.2 nm, which is quite similar to the ideal monolayer thickness of clay^36^. In contrast, the *H* value of PEG was found to be 5.5 nm. These results indicate that the C12P(17.5)- modified clay had peeled into single layers inside the PEG matrix.

The effects of the ring structure and the long alkyl chains of C12P5A on the nanocomposite structure were investigated using C2P(9.2) and UM(13.3) (Fig. 5c). The PXRD pattern of PEG + C2P(9.2) exhibited a peak at 2*θ* = 4.0° (*d*_001_ = 22 Å). The corresponding distance is larger than that observed for C2P(9.2) alone, but smaller than that of PEG + C12P(17.5). We assume that it is difficult for C2P5A inside the clay interlayer to form host–guest complexes. Because C2P5A has ten cationic moieties in close proximity to its ring structure, individual C2P5A units interact poorly in the bulk state. In addition, the strong interactions between the C2P5A units and the layered silicate made it difficult for the PEG molecules to penetrate the C2P5A cavity. The effect of the ring structure was investigated by mixing UM(13.3) and PEG, where the resultant PEG + UM(13.3) exhibited peaks at 2*θ* = 2.5° (*d*_001_ = 35 Å) derived from PEG and 2*θ* = 4.6° (*d*_001_ = 19 Å) derived from UM(13.3). This indicates that non-exfoliated clays are dispersed in the PEG. Furthermore, because PEG and UM cannot form host–guest complexes, insufficient energy was available to separate and peel the clay layers. Therefore, we concluded that clay exfoliation did not occur.

**Figure 6:**
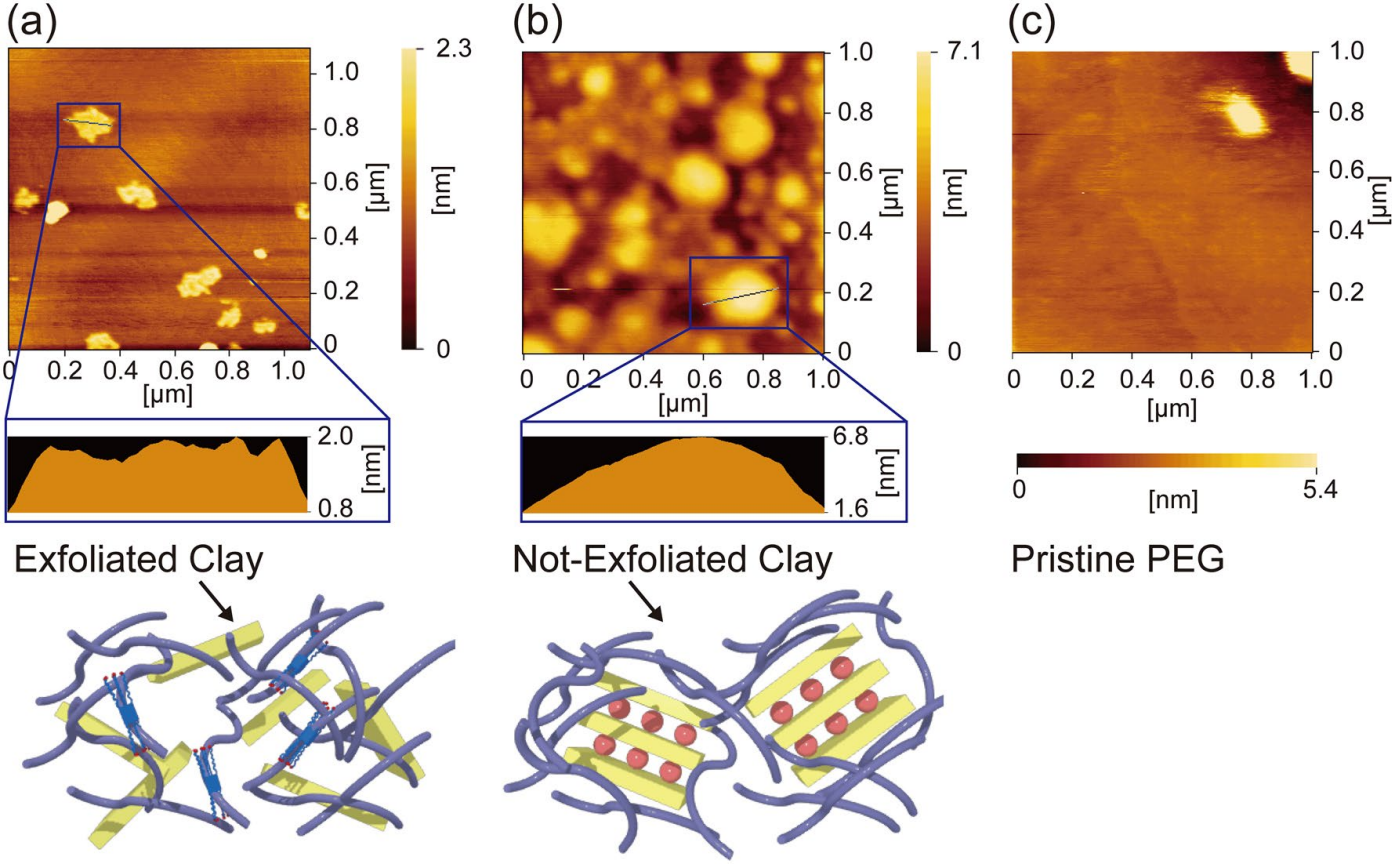
Clay morphology. Atomic force microscopy (AFM) images and altitude information for clay nanocomposites (**a**) with and (**b**) without C12P5A as the intercalant, and (**c**) PEG as the polymer matrix.

Atomic force microscopy (AFM) was used to evaluate and confirm that single-layer clays had formed during exfoliation. Films were prepared by casting methanolic solutions of composite materials onto mica substrates. PEG + C12P(17.5), pristine clay with PEG, and pristine PEG formed films on the substrates, with the corresponding nanoscale AFM images displayed in Fig. 6. Micro-order-sized structures were observed in PEG + C12P(17.5) and PEG + pristine, while the PEG film was homogeneous (Fig. 6c). Therefore, we concluded that the white PEG + C12(17.5) and PEG + pristine structures are clays dispersed in PEG matrices. We determined the sizes of these clay structures by depth profiling. The distances between the tops and bottoms of the white structures were determined as approximately 1.2 and 5.3 nm for PEG + C12P(17.5) and PEG + pristine, respectively (Fig. 6a, b). Because the pristine clay is 6.5 nm thick, we concluded that single-layer silicates were obtained using the C12P-modified clays; in fact, the value of 1.2 nm is quite similar to the thickness of an ideal monolayered clay.

## Discussion

From the results presented in the previous section, we evaluated the exfoliation behavior of clay in terms of host–guest interactions with the pillar[5]arene structure (Fig. 4). The cations present in the clay interlayer spaces interact with the anions of the layered silicate to form a multilayered structure. These interactions, which occur at multiple points, completely cancel the surface charges of the layers by introducing cations, including hydrated cations. These cations could be exchanged with other cations similar in size, valence, and hydration state. Therefore, this intercalation phenomenon, which typically involves organic and poly-cationic molecules exchanging their cations, causes the clay to disperse and improves its affinity toward the polymer matrix. The cationic intercalant prioritizes affinity with the polymer and cation uptake. Hence, it is extremely difficult to disperse clay in its single-layered form. Even in the bentonite/12-aminolaurate hydrochloride (ARA) combination, neither the large interlayer distance nor improvements in clay dispersibility and partial exfoliation were confirmed (Table 1). In contrast, C12P5A, which is a cyclic compound containing ten alkylammonium cationic moieties in a single molecule, was easily intercalated. It was also possible to increase the interlayer distance within the clay. The symmetrical cyclic structure of the pillar[5]arene facilitates interactions between its benzene rings and the alkyl chains. In addition, because C12P5A has ten cationic moieties, it interacts with the clay more strongly than the hydrated, alkaline earth or alkali cations initially present within the clay are able to. Therefore, the uptake of the bulky C12P5A resulted in an increase in the interlayer distance in the clay.

Host–guest interactions of pillar[5]arenes have been revealed by our group to not only occur in solution, but also in bulk^26,37,38^. From the interlayer distances determined by PXRD, the dense C12P5A is inclined to be present in the interlayer of the modified clay. Additionally, because bulk pillar[5]arene was present between the layers following intercalation, host–guest interactions led to the formation of polyrotaxanes with PEG. In fact, we used ^1^H NMR spectroscopy to confirm that pillar[5]arenes interact with PEG to form polyrotaxanes (Figure S9), which suggests that the host–guest interactions that form the polyrotaxane also drive polymer penetration between the layers to separate the layered structure.

To confirm that host–guest interactions are penetrative, we performed exfoliation experiments using adiponitrile as a competitive guest that strongly interacts with pillar[5]arene in a host–guest manner (Figure S10). Adiponitrile was added to C12P(17.5) before being mixed with PEG to form a pillar[5]arene inclusion complex. When the clay including the competitive guest was composited under similar conditions used for the exfoliated clay, the clay precipitated from PEG without exfoliation (Figure S11). In addition, composited materials using the clay modified with monomer units showed almost no change in their PXRD diffraction peaks (Fig. 3c). In addition, SAXS profiles were also obtained from the layered structure (Table 1). The Guinier plots of C2P(9.2)–PEG and UM(13.3)–PEG show slight deviations from the corresponding fitted curves, which is ascribed to the composites containing non-exfoliated clays. Based on these results, we conclude that host–guest interactions in which the polymer penetrates into the ring structure of the pillar[5]arene cause clay exfoliation.

## Conclusion

We clarified that host–guest interactions involving cationic pillar[5]arenes can be effectively used to exfoliate clay. There are many types of clay, and exfoliated clays exhibit a multitude of properties because of their many constituents. However, clay exfoliation is difficult to achieve because each layer is strongly adhesive. Cationic pillar[5]arenes can exfoliate many types of clay by exploiting host–guest interactions with PEG. We expect that the host–guest interactions of pillar[5]arenes will be effectively used to prepare exfoliated clay composites for applications such as electronic devices, mechanical materials, and biomaterials by providing a new method for forming composite materials.

## Methods

To prepare modified clays, bentonite (80 mg) and intercalants such as C12P5A, C2P5A and unit model of C12P5A (20 mg) were added with water (20 mL) in a 100 mL round bottom flask. The mixture was stirred at 80 °C under reflux for 18 h. After cooling to room temperature, the reacted mixture was centrifuged and the supernatant was removed to collect the precipitate. The precipitate was washed with excess water or methanol by centrifugation three times, respectively. Subsequently, water was added to the precipitate to collect precipitate by filtration. Then, the modified clay was dried in vacuo. For changing the mixing ratio, the intercalant was set to 5, 10, 20, 30, and 50 wt% with pristine clay.

The nanocomposite materials were prepared by poly(ethylene glycol) of 1 k molecular weight in bulk state. The modified clays (5 mg) were added to melted PEG (95 mg) at 80 °C. The mixture was stirred for 18 h to give nanocomposite materials.

## Supplementary Information

Supplementary Information.

## Data Availability

The authors declare that all data supporting the findings of this study are available within the Article and its Supplementary Information. The raw data generated in this study is available from the corresponding author upon reasonable request.

## References

[1] Durr UH, Gildenberg M, Ramamoorthy A (2012). The magic of bicelles lights up membrane protein structure. Chem. Rev..

[2] Eloy JO (2014). Liposomes as carriers of hydrophilic small molecule drugs: Strategies to enhance encapsulation and delivery. Colloids Surf. B Biointerfaces.

[3] Burns RA, Roberts MF, Dluhy R, Mendelsohn R (1982). Monomer-to-micelle transition of dihexanoylphosphatidylcholine: Carbon-13 NMR and Raman studies. J. Am. Chem. Soc..

[4] Altschuler ZS, Dwornik EJ, Kramer H (1963). Transformation of montmorillonite to kaolinite during weathering. Science.

[5] Endo M (1997). Stacking nature of graphene layers in carbon nanotubes and nanofibres. J. Phys. Chem. Solids.

[6] Nguyen VL (2020). Layer-controlled single-crystalline graphene film with stacking order via Cu-Si alloy formation. Nat. Nanotechnol..

[7] Marshall CE, Caldwell OG (1947). The colloid chemistry of the clay mineral attapulgite. J. Phys. Chem..

[8] Zhang Y (2018). Temperature induced interface and optical properties of the multi-layer nanotube network. J. Appl. Phys..

[9] Jin Q (2011). Self-assembly of copper(II) ion-mediated nanotube and its supramolecular chiral catalytic behavior. Langmuir.

[10] Vasiljevic J (2020). Characterization of polyamide 6/multilayer graphene nanoplatelet composite textile filaments obtained via in situ polymerization and melt spinning. Polymers (Basel).

[11] Paul DR, Robeson LM (2008). Polymer nanotechnology: Nanocomposites. Polymer.

[12] Kawasumi M, Hasegawa N, Usuki A, Okada A (1998). Nematic liquid crystal/clay mineral composites. Mater. Sci. Eng. C.

[13] Bezrodna T (2005). Effects of montmorillonite modification on optical properties of heterogeneous nematic liquid crystal–clay mineral nanocomposites. Liq. Cryst..

[14] Baran J (2007). Effect of clay surface modification on the structure and electro-optical properties of liquid crystal/clay nanocomposites. Philos. Mag..

[15] Jlassi K (2014). Exfoliated clay/polyaniline nanocomposites through tandem diazonium cation exchange reactions and in situ oxidative polymerization of aniline. RSC Adv..

[16] Kuroda K (1999). An acentric arrangement of p-nitroaniline molecules between the layers of kaolinite. Chem. Commun..

[17] Hensen EJM, Smit B (2002). Why clays swell. J. Phys. Chem. B.

[18] Begg JD, Edelman C, Zavarin M, Kersting AB (2018). Sorption kinetics of plutonium (V)/(VI) to three montmorillonite clays. Appl. Geochem..

[19] Dasari A, Yu Z, Mai Y, Hu G, Varlet J (2005). Clay exfoliation and organic modification on wear of nylon 6 nanocomposites processed by different routes. Compos. Sci. Technol..

[20] Katoh Y, Okamoto M (2009). Crystallization controlled by layered silicates in nylon 6–clay nano-composite. Polymer.

[21] Mizuno C, John B, Okamoto M (2013). Percolated network structure formation and rheological properties in nylon 6/Clay nanocomposites. Macromol. Mater. Eng..

[22] Akita I, Ishida Y, Yonezawa T (2020). Distinctive stability of a free-standing monolayer clay mineral nanosheet via transmission electron microscopy. Phys. Chem. Chem. Phys..

[23] Michels-Brito PH (2021). Unmodified clay nanosheets at the air-water interface. Langmuir.

[24] Ogoshi T, Kakuta T, Yamagishi TA (2019). Applications of pillar[n]arene-based supramolecular assemblies. Angew. Chem. Int. Ed..

[25] Kakuta T, Yamagishi TA, Ogoshi T (2018). Stimuli-responsive supramolecular assemblies constructed from pillar[n]arenes. Acc. Chem. Res..

[26] Ogoshi T (2019). Molecular weight fractionation by confinement of polymer in one-dimensional pillar[5]arene channels. Nat. Commun..

[27] Nam PH (2001). A hierarchical structure and properties of intercalated polypropylene/clay nanocomposites. Polymer.

[28] Ogoshi T, Sueto R, Yoshikoshi K, Yasuhara K, Yamagishi TA (2016). Spherical vesicles formed by co-assembly of cyclic pentagonal pillar[5]quinone with cyclic hexagonal pillar[6]arene. J. Am. Chem. Soc..

[29] Ogoshi T, Takashima S, Yamagishi TA (2015). Molecular recognition with microporous multilayer films prepared by layer-by-layer assembly of pillar[5]arenes. J. Am. Chem. Soc..

[30] Ogoshi T (2019). Guest vapor-induced state change of structural liquid pillar[6]arene. J. Am. Chem. Soc..

[31] Ogoshi T (2018). Ring shape-dependent self-sorting of pillar[n]arenes assembled on a surface. Commun. Chem..

[32] Kohay H (2017). PEG-PE/clay composite carriers for doxorubicin: Effect of composite structure on release, cell interaction and cytotoxicity. Acta Biomater..

[33] Masa A, Saito H, Sakai T, Kaesaman A, Lopattananon N (2017). Morphological evolution and mechanical property enhancement of natural rubber/polypropylene blend through compatibilization by nanoclay. J. Appl. Polym. Sci..

[34] Renouprez, A. J. in *Catalyst Characterization: Physical Techniques for Solid Materials* (eds Boris Imelik & Jacques C. Vedrine), 445–465 (Springer US, New York, 1994).

[35] Shang C, Rice JA (2001). Interpretation of small-angle x-ray scattering data from dilute montmorillonite suspensions using a modified Guinier approximation. Phys. Rev. E.

[36] Khoeini M, Bazgir S, Tamizifar M, Nemati A, Arzani K (2009). Investigation of the modification process and morphology of organosilane modified nanoclay. Ceramics-Silikáty.

[37] Ogoshi T (2018). Separation of linear and branched alkanes using host-guest complexation of cyclic and branched alkane vapors by crystal state pillar[6]arene. Angew. Chem. Int. Ed..

[38] Ogoshi T (2017). Alkane-length sorting using activated pillar[5]arene crystals. Chem. Commun..

