# Supplementary material for: Supramolecular exfoliation of layer silicate clay by novel cationic pillar[5]arene intercalants
Source: Sci Rep. 2021 May 20;11:10637. doi: 10.1038/s41598-021-90122-9 (PMC8137868; doi:10.1038/s41598-021-90122-9)
Supplement: Supplementary file 1 — Supplementary Information. [file 41598_2021_90122_MOESM1_ESM.docx]

Supplementary Information

Supramolecular Exfoliation of Layer Silicate Clay

by Novel Cationic Pillar[5]arene Intercalants

Takahiro Kakuta,^a,b^ Yudai Baba,^a^ Tada-aki Yamagishi^a^ and Tomoki Ogoshi*^b,c^

^a.^Graduate School of Natural Science and Technology, Kanazawa University, Kakuma-machi, Kanazawa 920-1192, Japan.

^b.^ WPI Nano Life Science Institute (WPI-NanoLSI), Kanazawa University, Kakuma-machi, Kanazawa 920-1192, Japan.

^c.^Graduate School of Engineering, Kyoto University, Katsura, Nishikyo-ku, Kyoto, 615-8510, Japan.

**Supplementary Information**

**Table of Contents**

1. Materials and Measurements
2. Synthesis of dodecyl triethylamine functionalized pillar[5]arene (C12P5A)
   1. **Figure S1.** ^1^H NMR spectrum of C12P5A (CHCl_3_-*d*, 500 MHz, 303 K).
   2. **Figure S2.** ^13^C NMR spectrum of C12P5A (CHCl_3_-*d*, 125 MHz, 303 K).
3. Synthesis of monomer unit of C12P5A (UM)
   1. **Figure S3.** ^1^H NMR spectrum of modified clay with the C12P5A molecular unit (**UM**) (MeOH-*d*_3_, 500 MHz, 303 K).
   2. **Figure S4.** ^13^C NMR spectrum of UM (MeOH-*d*_3_, 125 MHz, 303 K).
4. Synthesis of ethyl triethylamine-functionalized pillar[5]arene (C2P5A)
   1. **Figure S5.** ^1^H NMR spectrum of C2P5A (MeOH-*d*_3_, 500 MHz, 303 K).
   2. **Figure S6.** ^13^C NMR spectrum of C2P5A (DMSO-*d*_6_, 125 MHz, 303 K).
5. Introduced amount of intercalants identified by TGA
   1. **Table S1.** Introduced amount of intercalants in modified clay.
6. SAXS patterns of composite materials
   1. **Figure S7.** Guinier plots of (a) C12P(17.5)–poly(ethylene glycol) (PEG), (b) C2P(9.2)–PEG, and (c) UM(13.3)–PEG.
   2. **Figure S8.** Proposed structures of (PEG) nanocomposites with (a) C12P, (b) C2P, and (c) UM.
7. Investigation of polyrotaxane formation
   1. **Figure S9.** ^1^H NMR spectrum of polyrotaxane (CHCl_3_-*d*, 500 MHz, 303 K).
8. Competitive experiments using adiponitrile
   1. **Figure S10.** Host–guest interaction of C12P5A with adiponitrile observed by ^1^H NMR (D_2_O, 500 MHz, 303 K).
   2. **Figure S11.** Guinier plots of composite materials composed of PEG and C12P(17.5) with adiponitrile.
9. Particle size analysis by simple Scherrer formula
   1. **Figure S12.** The particle size of clay after intercalation depending on (a) the percentage of C12P5A and (b) the type of intercalants.
10. Distribution of the clay thickness determined by AFM
    1. **Figure S13.** Thickness distribution of the clay by AFM.
11. SAXS patterns of PEG as a matrix polymer
    1. **Figure S14.** Guinier plots of PEG materials.

**1. Materials and Measurements**

**Materials**

All solvents and reagents were used as supplied without additional purification. We obtained bentonite and polyethylene glycol (PEG) from FUJIFILM Wako Pure Chemical Corporation. Sodium sulfate (Na_2_SO_4_), paraformaldehyde and ethyl acetate were obtained from Nacalai Tesque Inc. Hydroquinone, 1,12-dibromododecane, 1,2-dibromoethane, adiponitrile, trimethyl amine・EtOH and BF_3_–ethyl ether complex were obtained from Tokyo Chemical Industry Co., Ltd. Dichloromethane (CH_2_Cl_2_), 1,2-dichloroethane, methanol (CH_3_OH), chloroform (CHCl_3_), potassium carbonate (K_2_CO_3_), CHCl_3_-*d*, dimethyl sulfoxide (DMSO)-*d*_6_, deuterium oxide (D_2_O), and silica gel were obtained from Kanto Chemical Co., Inc. Deionized water was purified by G series cartridge water purifiers (Organo Corporation).

**Measurements**

Solution ^1^H NMR spectra were recorded at 500 MHz with a JEOL‐ECA500 spectrometer. Solution ^13^C NMR spectra were recorded at 125 MHz with a JEOL‐ECA500 spectrometer. UV-Vis reflection spectra were measured by a JASCO V‐670 spectrometer with ISN-723. Thermogravimetric analysis (TGA) data were recorded at 10 °C/min under air by a SII-TG/DTA6200 system with a platinum pan. Powder X‐ray diffraction (PXRD) measurements were performed on a Rigaku-SmartLab high-resolution diffractometer using monochromated CuKα_1_ radiation (λ = 1.5406 Å). Small angle X-ray scattering (SAXS) measurements were performed on a Rigaku- Nano Viewer (RA-MICRO 7HFM). Atomic force microscopy (AFM) measurements were performed on a SII-Nanocute with a micro cantilever (SI-DF20).

**2. Synthesis of dodecyl triethylamine functionalized pillar[5]arene (C12P5A)**

・

**C12P5A**

**2**

**1**

Hydroquinone (1.00 g, 9.00 mmol) and 1,12-dibromododecane (44.3 g, 137 mmol) were dissolved in acetone (200 mL). Subsequently, anhydrous K_2_CO_3_ (3.77 g, 27.3 mmol) was reacted under reflux for 24 h. The reaction mixture was filtered, and the filtrate was evaporated under reduced pressure. The crude product was extracted with CH_2_Cl_2_ before washing with brine. The organic layer was dried with Na_2_SO_4_ before removing the solvent under reduced pressure. Recrystallization from CH_2_Cl_2_ and CH_3_OH gave **1** as a white solid (2.70 g, 4.45 mmol, 49%). ^1^H NMR (500 MHz, CHCl_3_-*d*), *δ* (TMS, 0 ppm) : 6.96-6.90 (d, 4*H*, phenyl H), 3.9-3.88 (t, 4*H*, C_12_H_24_), 3.43-3.40 (t, 4*H*, C_12_H_24_ ), 1.85-1.83 (m, 4*H*, C_12_H_24_), 1.77-1.75 (m, 4*H*, C_12_H_24_), 1.45-1.30 (m, 32*H*, C_12_H_24_).

Compound **1** (500 mg, 0.827 mmol) and paraformaldehyde (76 mg, 2.50 mmol) were dissolved in dichloroethane (30 mL). The mixture was stirred at 0 °C and then boron trifluoride diethyl etherate (BF_3_·O(C_2_H_5_)_2_, 0.15 mL, 1.03 mmol) was added to the solution and the mixture was stirred at room temperature for 5 h. The reaction mixture was quenched by CH_3_OH (50 mL) and the precipitate was collected by filtration. The crude product was purified by silica gel column chromatography (hexane/ CH_2_Cl_2_ = 7/3) affording **2** as a white solid (118 mg, 0.038 mmol, 23%). ^1^H NMR (500 MHz, CHCl_3_-*d*), *δ* (TMS, 0 ppm) : 6.92-6.90 (s, 10*H*, phenyl H), 3.95-3.92 (t, 20*H*, C_12_H_24_), 3.78-3.74 (t, 20*H*, C_12_H_24_), 3.35-3.32 (s, 10*H*, CH_2_), 1.85-1.83 (m, 20*H*, C_12_H_24_), 1.77-1.75 (m, 20*H*, C_12_H_24_), 1.45-1.30 (m, 128*H*, C_12_H_24_).

Compound **2** (100 mg, 0.0320 mmol) and trimethylamine in ethanol (2.00 mL, 3.20 mmol) were reacted under reflux for 24 h. The reaction mixture was washed with CHCl_3_, and aqueous layer was evaporated under reduced pressure affording C12P5A as a white crystalline solid (112 mg, 0.0310mmol, 97%). ^1^H NMR (500 MHz, DMSO-*d*_6_), *δ* (TMS, 0 ppm): 6.92-6.90 (s, 10*H*, phenyl H), 3.93-3.91 (t, 20*H*, C_12_H_24_), 3.72-3.70 (t, 20*H*, C_12_H_24_), 3.63-3.60 (s, 10*H*, CH_2_), 3.10-3.08 (s, 110*H*, N(CH_3_)_3_, C_12_H_24_), 1.80-1.62 (m, 20*H*, C_12_H_24_), 1.56-1.48 (m, 20*H*, C_12_H_24_), 1.45-1.28 (m, 128*H*, C_12_H_24_). ^13^C NMR (DMSO-*d*_6_, 125 MHz), δ (TMS, 0 ppm): 29.71, 30.00, 52.64, 65.68, 114.44, 128.88, 149.63. HRMS (ESI) calcd. for C_185_H_350_N_10_O_10_Br_10_ [M-2Br]^2+^: 1756.5297, found 1756.6414; [M-3Br]^3+^: 1144.3820, found 1144.3913; [M-4Br]^4+^: 838.3059, found 838.3054; [M-5Br]^5+^: 654.4614, found 654.4607; [M-6Br]^6+^: 532.2313, found 532.2314; [M-7Br]^7+^: 444.7821, found 444.4720; [M-8Br]^8+^: 379.1940, found 379.1947; [M-9Br]^9+^: 328.1821, found 328.1820; [M-10Br]^10+^: 287.3717, found 287.3720.


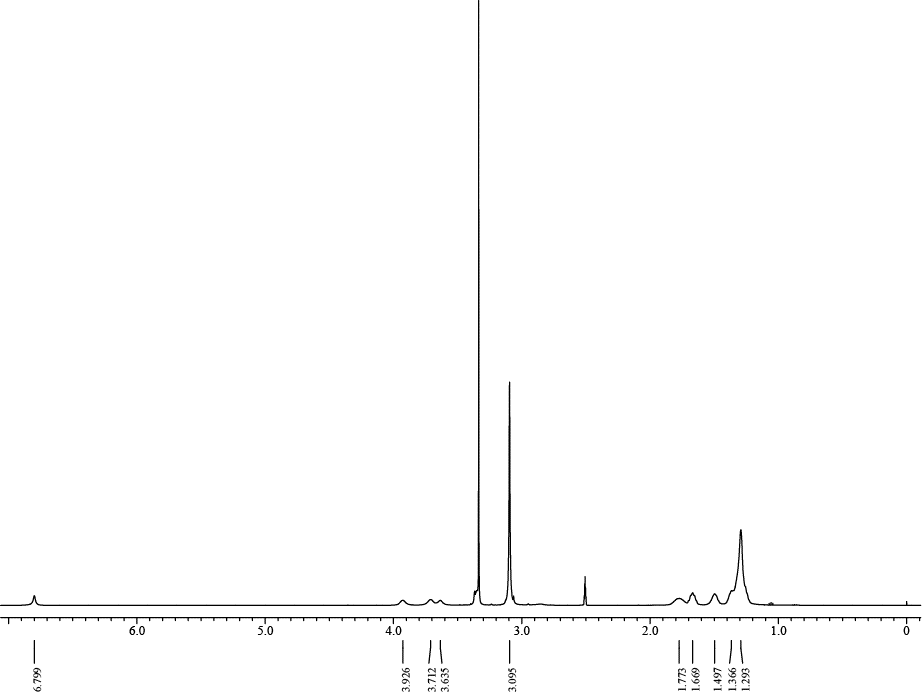


HDO

DMSO

ppm

**Figure S1.** ^1^H NMR spectrum of C12P5A (DMSO-*d*6, 500 MHz, 303 K).


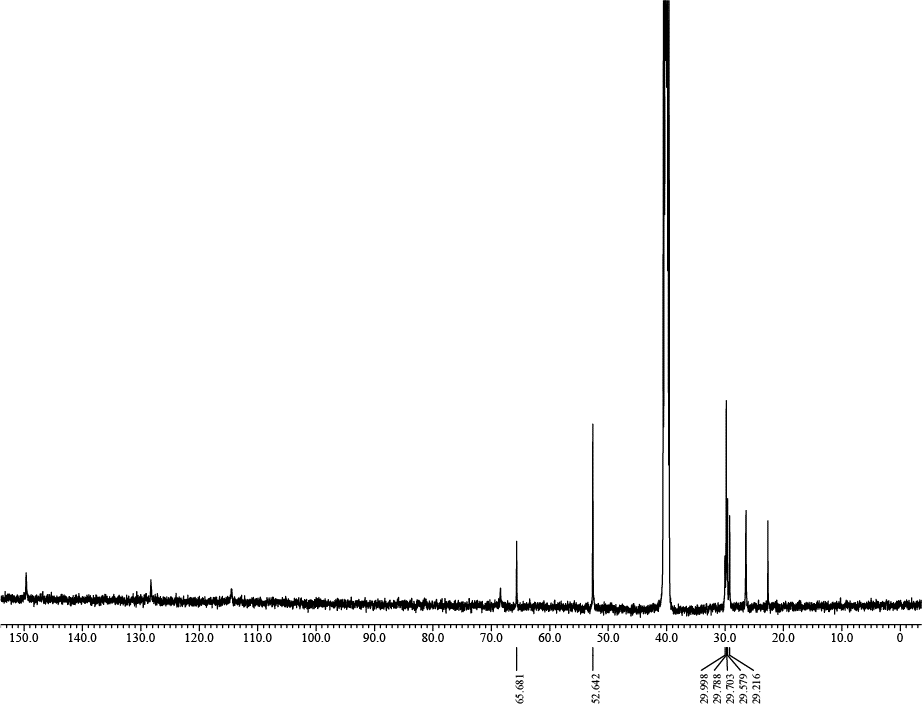


ppm

**Figure S2.** ^13^C NMR spectrum of C12P5A (DMSO-*d*6, 125 MHz, 303 K).

**3. Synthesis of monomer unit of C12P5A (UM)**

**C12P5A (UM)**

**1**

Compound **1** (100 mg, 0.016 mmol) and trimethylamine in ethanol (1 mL, 1.6 mmol) were reacted under reflux for 24 h. The reaction mixture was washed with CHCl_3_, and aqueous layer was evaporated under reduced pressure affording a modified clay with the C12P5A molecular unit (**UM**) as a white crystalline solid (108 mg, 0.149 mmol, 93%). ^1^H NMR (500 MHz, MeOH-*d*_3_), *δ* (TMS, 0 ppm): 6.81 (s, 4*H*, phenyl H), 3.85-3.92 (t, 4*H*, C_12_H_24_), 3.10-3.15 (s, 22*H*, N(CH_3_)_3_, C_12_H_24_), 1.74-1.72 (m, 4*H*, C_12_H_24_), 1.78-1.74 (m, 4*H*, C_12_H_24_), 1.44-1.33 (m, 32*H*, C_12_H_24_). ^13^C NMR (MeOH-*d*_3_, 125 MHz), δ (TMS, 0 ppm): 22.62, 25.85, 26.06, 28.82, 29.19, 52.21, 66.57, 68.32, 115.1, 153.3.


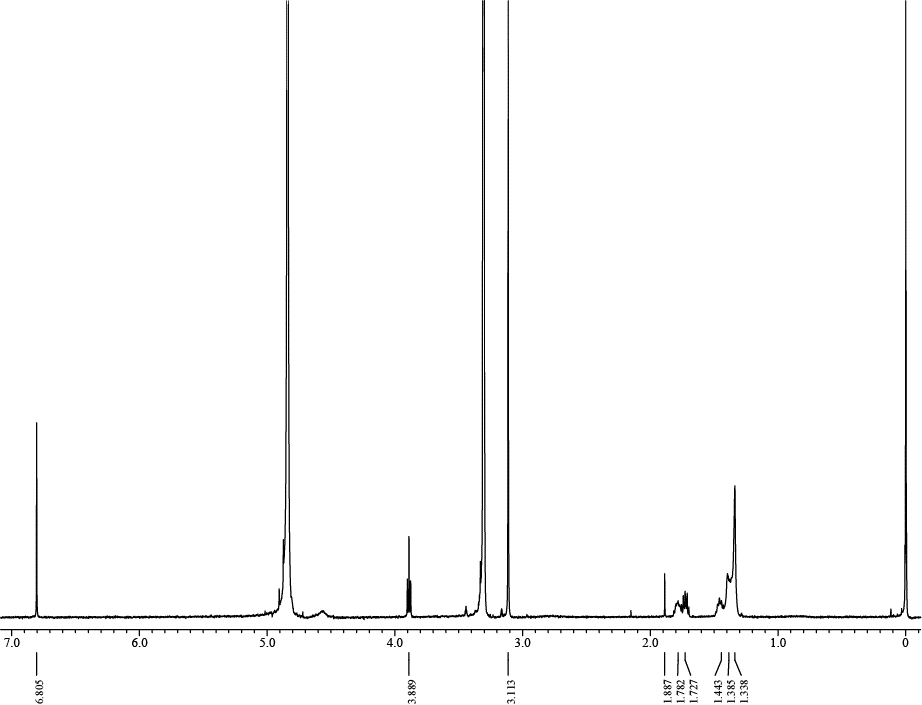


CD_3_OH

HDO

ppm

**Figure S3.** ^1^H NMR spectrum of modified clay with the C12P5A molecular unit (**UM**) (MeOH-*d*_3_, 500 MHz, 303 K).


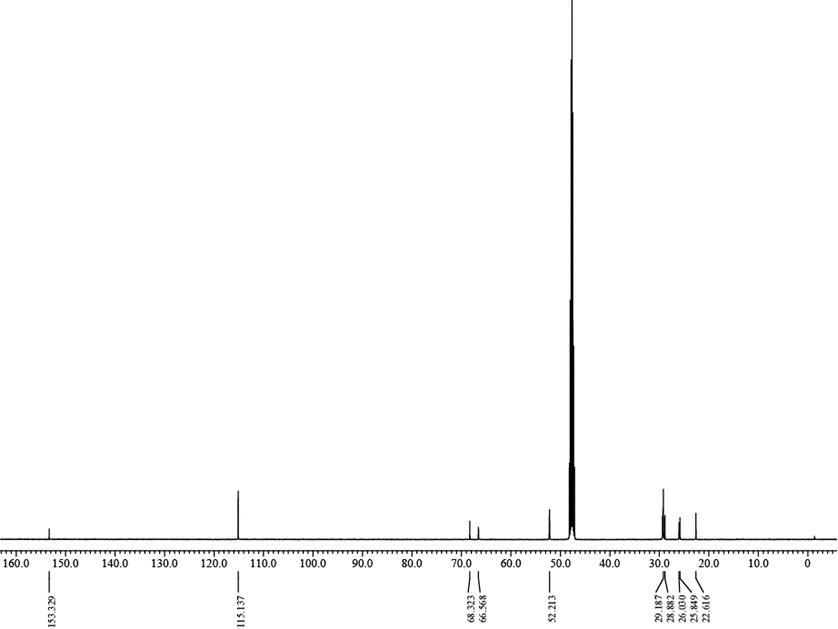


ppm

**Figure S4.** ^13^C NMR spectrum of **UM** (MeOH-*d*_3_, 125 MHz, 303 K).

**4. Synthesis of ethyl trimethylamine-functionalized pillar[5]arene (C2P5A)**

**C2P5A**

・

**4**

**3**

C2P5A was synthesized according to a previous paper.^S1^

Hydroquinone (1.00 g, 9.00 mmol) and 1,2-dibromoethane (6.76 g, 36.0 mmol) were dissolved in acetone (200 mL). Subsequently, anhydrous K_2_CO_3_ (3.77 g, 27.3 mmol) was added under reflux before stirring for 24 h. The reaction mixture was filtered, and the filtrate was evaporated under reduced pressure. The crude product was extracted with CH_2_Cl_2_ before washing with brine. The organic layer was dried with Na_2_SO_4_ and removed under reduced pressure. Recrystallization from CH_2_Cl_2_ and CH_3_OH gave **3** as a white solid (1.31 g, 4.05 mmol, 45%). ^1^H NMR (500 MHz, CHCl_3_-*d*), *δ* (TMS, 0 ppm) : 6.90-6.85 (d, 4*H*, phenyl H), 4.3-4.2 (t, 4*H*, C_2_H_4_), 3.65-3.60 (t, 4*H*, C_2_H_4_ ).

Compound **3** (500 mg, 1.54 mmol) and paraformaldehyde (140 mg, 4.62 mmol) were dissolved in dichloroethane (60 mL). The mixture was stirred at 0 °C and then BF_3_·O(C_2_H_5_)_2_ (0.25 mL, 1.71 mmol) was added to the solution and it was stirred at room temperature for 5 h. The reaction mixture was quenched by CH_3_OH (90 mL) and was filtered to collect the precipitate. The crude product was purified by silica gel column chromatography (hexane/CH_2_Cl_2_ = 7/3), affording **4** as a white solid (165 mg, 0.098 mmol, 32% ). ^1^H NMR (500 MHz, CHCl_3_-*d*), *δ* (TMS, 0 ppm) : 6.92-6.90 (s, 10*H*, phenyl H), 4.25-3.20 (t, 20*H*, C_2_H_4_), 3.65-3.60 (t, 20*H*, C_12_H_24_), 3.9-3.8 (s, 10*H*, CH_2_).

Compound **4** (100 mg, 0.060 mmol) and trimethylamine in ethanol (0.36 mL, 0.60 mmol) were reacted under reflux for 24 h. The reaction mixture was washed with CHCl_3_, and the aqueous layer was evaporated under reduced pressure, affording **C2P5A** as a white crystalline solid (132 mg, 0.0580 mmol, 98%). ^1^H NMR (500 MHz, MeOH-*d*_3_), *δ* (TMS, 0 ppm): 7.02-6.94 (s, 10*H*, phenyl H), 4.55-4.45 (t, 20*H*, C_12_H_24_), 4.14-4.00 (t, 20*H*, C_12_H_24_), 3.92-3.85 (s, 10*H*, CH_2_), ^13^C NMR (DMSO-*d*_6_, 125 MHz), δ (TMS, 0 ppm): 53.70, 63.03, 65.05, 116.05, 128.54, 149.30.

S1: Ma, Y. *et al.* A cationic water-soluble pillar[5]arene: synthesis and host–guest complexation with sodium 1-octanesulfonate. *Chem. Commun.* **47**, 12340-12342, doi:10.1039/C1CC15660H (2011).


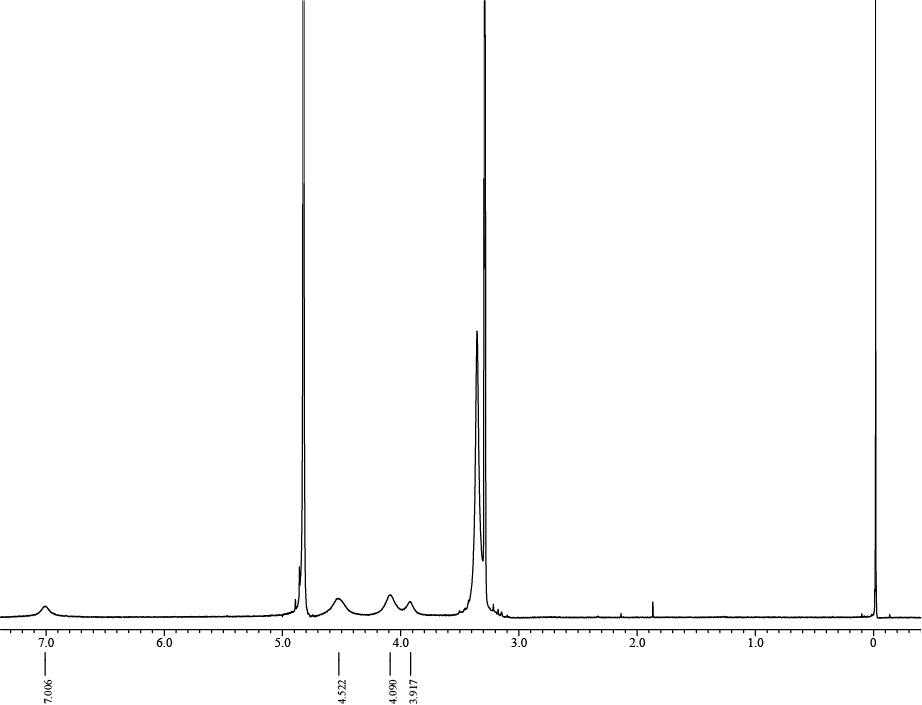


HDO

CD_3_OH

ppm

**Figure S5.** ^1^H NMR spectrum of **C2P5A** (MeOH-*d*_3_, 500 MHz, 303 K).


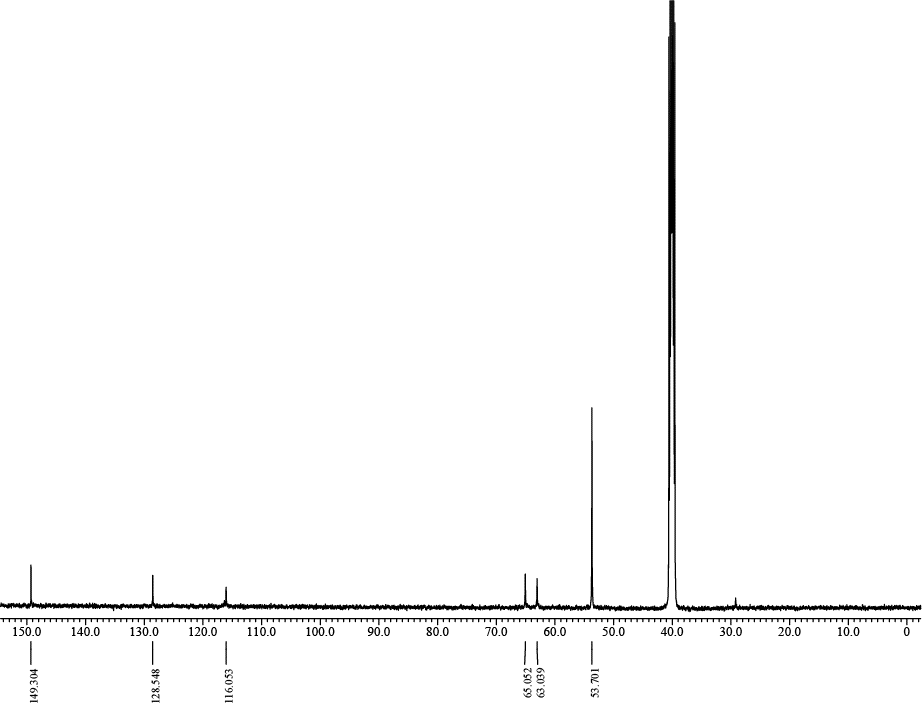


ppm

**Figure S6.** ^13^C NMR spectrum of **C2P5A** (DMSO-*d*_6_, 125 MHz, 303 K).

**5. Introduced amount of intercalants identified by TGA**

**Table S1.** Introduced amount of intercalants in modified clay.

| Sample | Intercalants | Feed ratio / wt% | R_c_ / wt% | R_dc_ / wt% | IC / wt% |
| --- | --- | --- | --- | --- | --- |
| Pristine Clay | - | - | 93.8 | - | - |
| C12P(X) | C12P5A | 5 |  | 89.1 | 4.7 |
|  |  | 10 |  | 85.0 | 8.8 |
|  |  | 20 |  | 76.3 | 17.5 |
|  |  | 30 |  | 78.1 | 15.7 |
|  |  | 50 |  | 75.8 | 18.0 |
| C2P(X) | C2P5A | 5 |  | 90.1 | 3.7 |
|  |  | 20 |  | 84.6 | 9.2 |
|  |  | 50 |  | 81.1 | 12.7 |
| UM(X) | UM | 5 |  | 90.1 | 3.7 |
|  |  | 20 |  | 80.5 | 13.3 |
|  |  | 50 |  | 78.8 | 15.0 |

**6. SAXS patterns of composite materials**


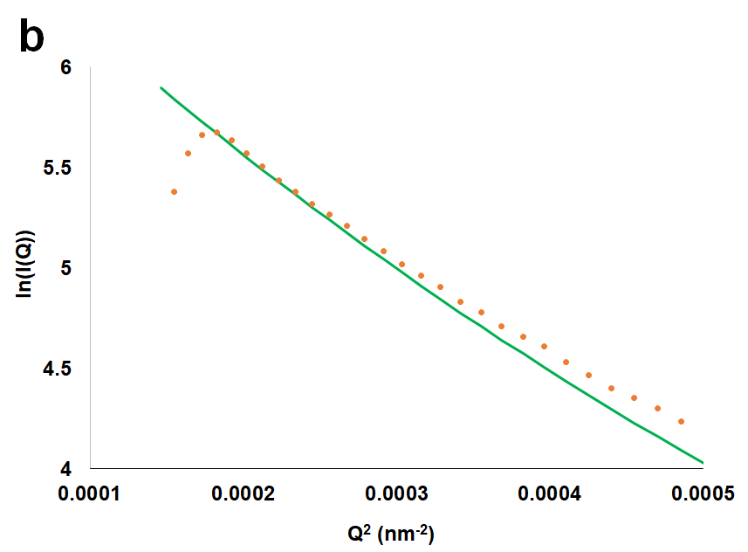

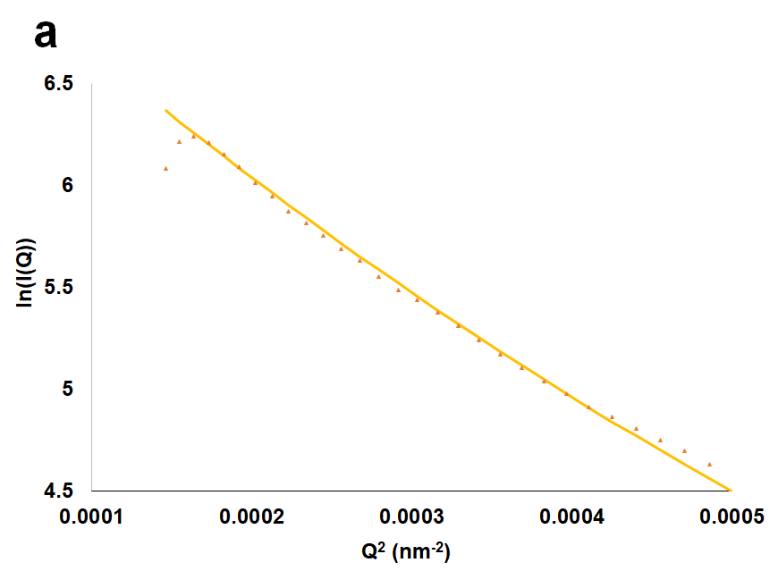


*R* = 5.7 nm

*R* = 5.4 nm


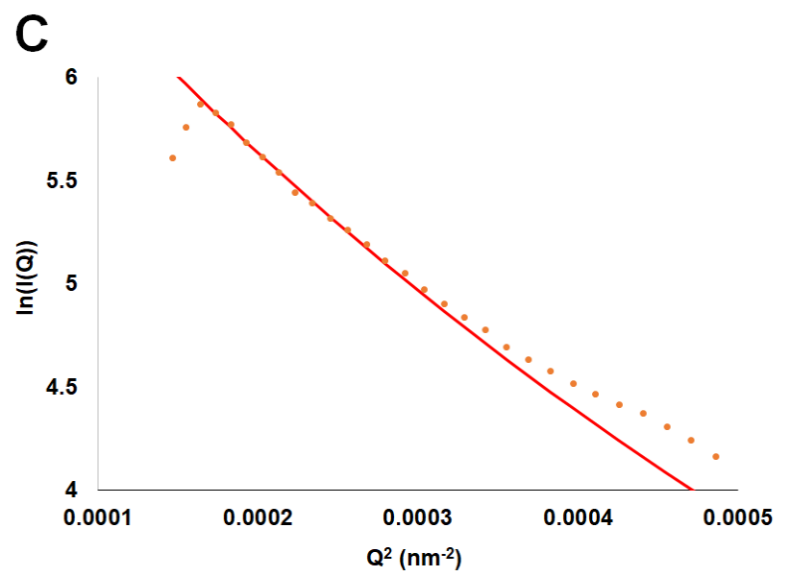


*R* = 6.2 nm

**Figure S7.** Guinier plots of (a) C12P(17.5)–poly(ethylene glycol) (PEG), (b) C2P(9.2)–PEG, and (c) UM(13.3)–PEG.


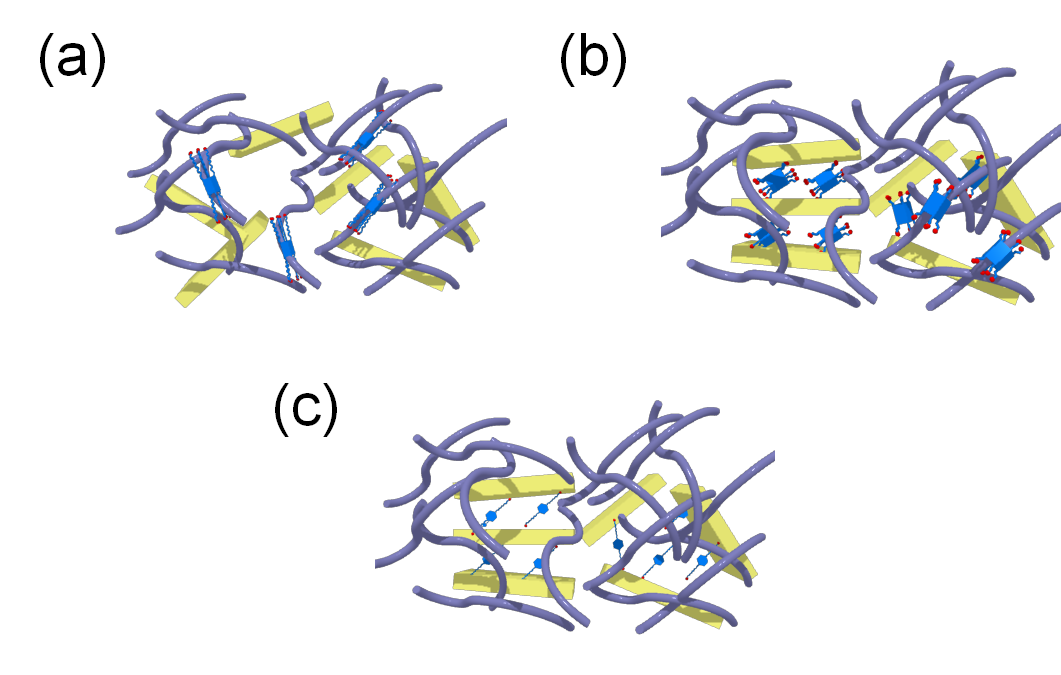


**Figure S8.** Proposed structures of PEG nanocomposites with (a) C12P, (b) C2P, and (c) UM.

**7. Investigation of polyrotaxane formation**


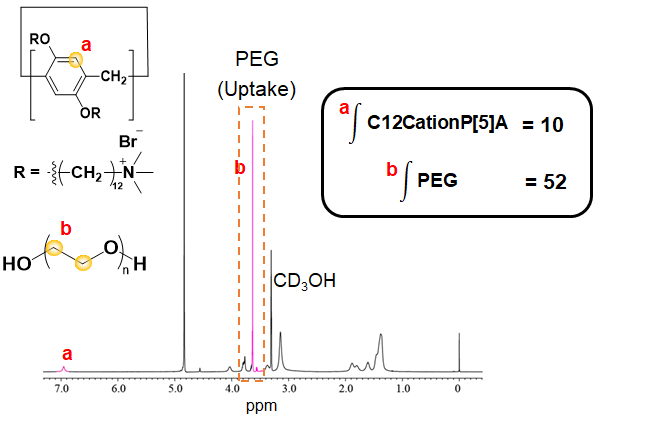


**Figure S9.** ^1^H NMR spectrum of polyrotaxane (MeOH-*d*_3_, 500 MHz, 303 K). PEG and C12P5A were added in glass vial and stirred at 80 °C for 18 h. In this case, an excess amount of PEG was used relative to the amount of C12P5A. The mixture was washed with CH_2_Cl_2_ to remove excess PEG and dried under the reduced pressure. The thirteen PEG units were encapsulated with one C12P5A.

**8. Competitive experiments using adiponitrile**


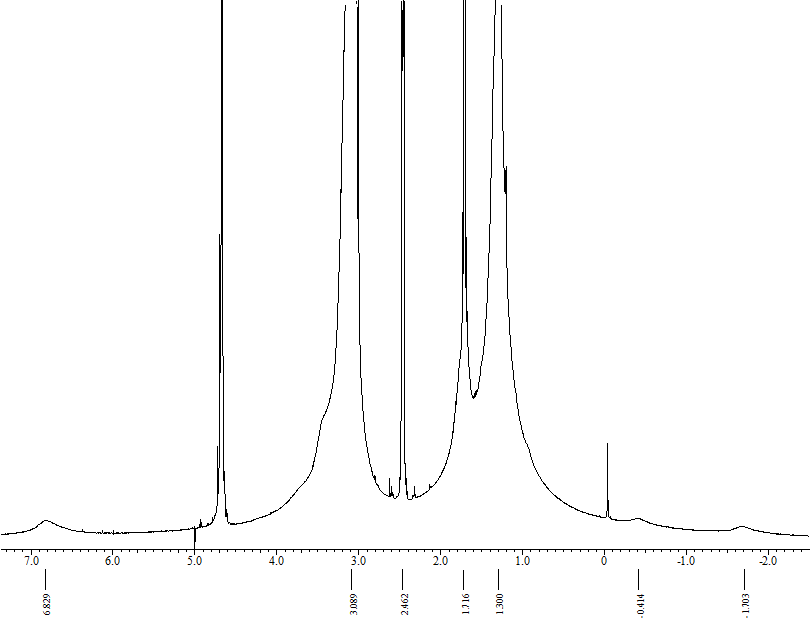


HDO

**a**

**a**

**b**

**b**

**adiponitrile**

a

b

ppm

**Figure S10.** Host–guest interaction of C12P5A with adiponitrile observed by ^1^H NMR (D_2_O, 500 MHz, 303 K). The sample was prepared so that the ratio of C12P5A/adiponitrile was 1/1.


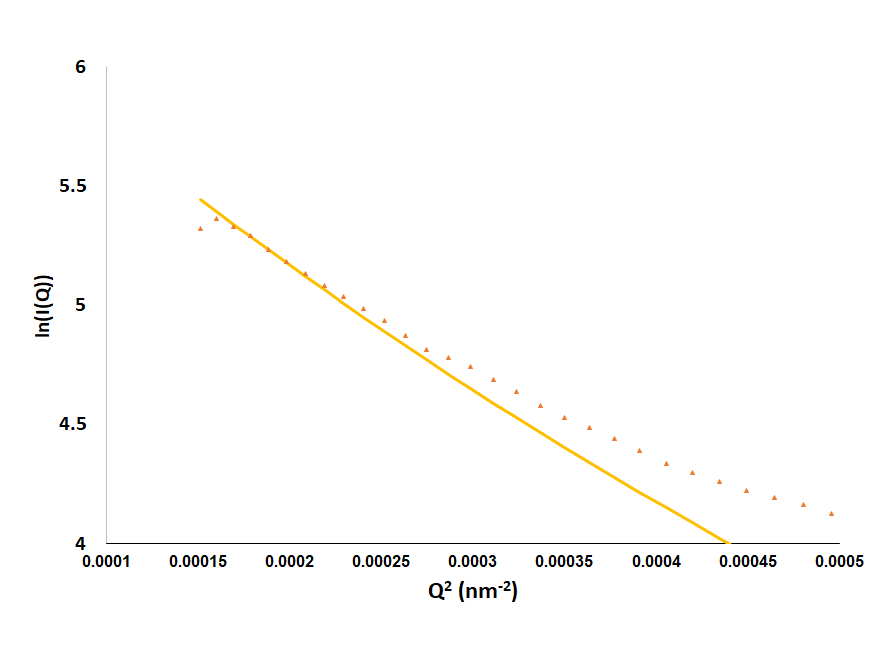


*R* = 5.7 nm

*H* = 5.5 nm

Aspect Ratio = 3.40

**Figure S11.** Guinier plot of composite materials composed of PEG and C12P(17.5) with adiponitrile.

C12P(17.5) (30 mg) and adiponitrile (0.5 mL, excess amount) were added in water under reflux and stirred for 18 h. The mixture was purified by centrifugation three times. Then, composite materials composed of PEG and C12P(17.5) with adiponitrile were prepared by mixing PEG and C12P(17.5) with adiponitrile at 80 °C.

**9. Particle size analysis by simple Scherrer formula**


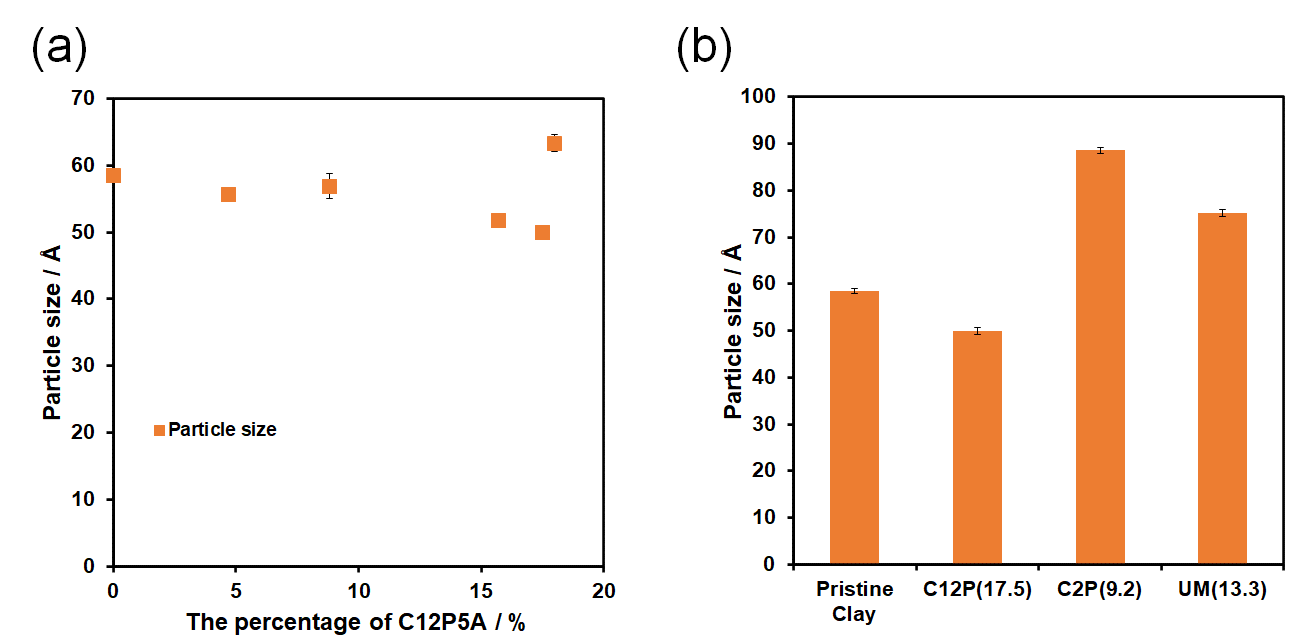


**Figure S12.** The particle size of clay after intercalation depending on (a) the percentage of C12P5A and (b) the type of intercalants.

S2: Michels, L. *et al.* The Impact of Thermal History on Water Adsorption in a Synthetic Nanolayered Silicate with Intercalated Li^+^ or Na^+^. *The Journal of Physical Chemistry C* **124**, 24690-24703, doi:10.1021/acs.jpcc.0c05847 (2020).

**10. Distribution of the clay thickness determined by AFM**


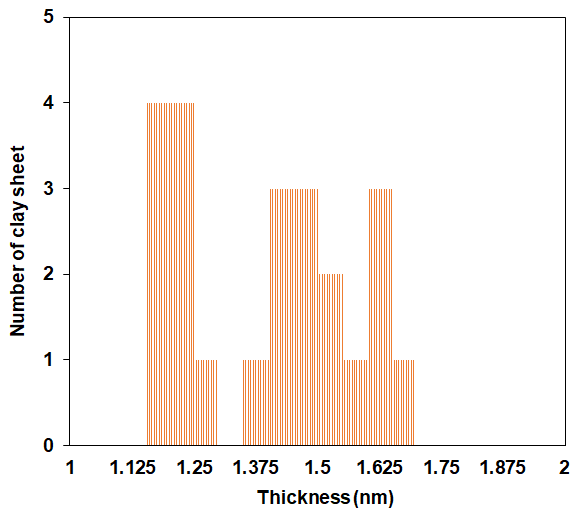


**Figure S13.** Thickness distribution of the clay determined by AFM.

**11. SAXS patterns of PEG as a matrix polymer**


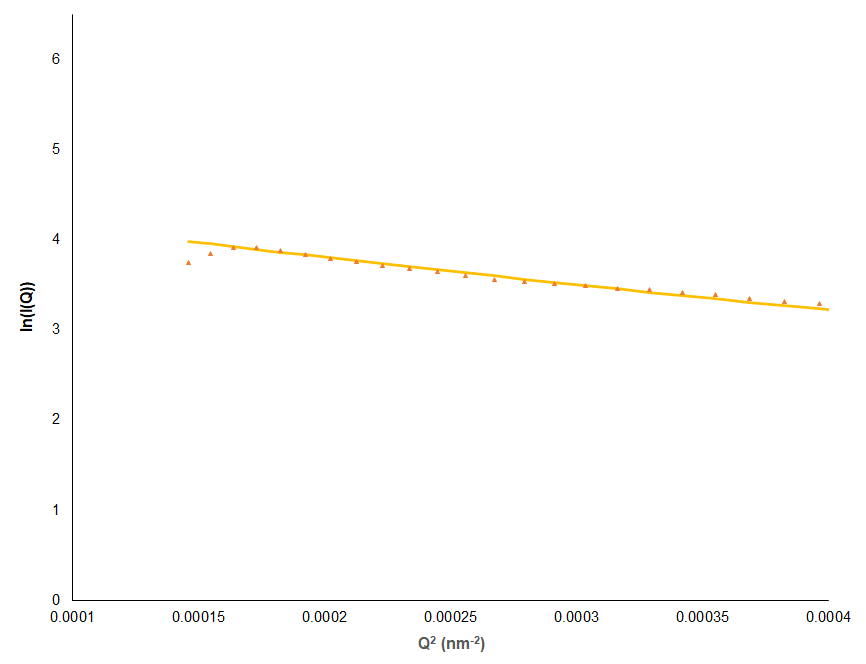


Aspect Ratio = 1.25

*R* = 5.0 nm

*H* = 5.5 nm

**Figure S14.** Guinier plots of PEG materials.
